# A Role for CD81 and Hepatitis C Virus in Hepatoma Mobility

**DOI:** 10.3390/v6031454

**Published:** 2014-03-24

**Authors:** Claire L. Brimacombe, Garrick K. Wilson, Stefan G. Hübscher, Jane A. McKeating, Michelle J. Farquhar

**Affiliations:** 1 Viral Hepatitis Research Group, Institute for Biomedical Research, University of Birmingham, Birmingham B15 2TT, UK; E-Mails: C.L.Brimacombe@bham.ac.uk (C.L.B.); G.K.Wilson@bham.ac.uk (G.K.W.); J.A.McKeating@bham.ac.uk (J.A.M.); 2 Centre for Liver Research and NIHR Birmingham Liver Biomedical Research Unit, Institute for Biomedical Research, University of Birmingham, Birmingham B15 2TT, UK; E-Mail: S.G.Hubscher@bham.ac.uk; 3 Department of Cellular Pathology, Queen Elizabeth Hospital Birmingham, Birmingham B15 2WB, UK

**Keywords:** hepatitis, tetraspanin, CD81, hepatoma, metastasis

## Abstract

Tetraspanins are a family of small proteins that interact with themselves, host transmembrane and cytosolic proteins to form tetraspanin enriched microdomains (TEMs) that regulate important cellular functions. Several tetraspanin family members are linked to tumorigenesis. Hepatocellular carcinoma (HCC) is an increasing global health burden, in part due to the increasing prevalence of hepatitis C virus (HCV) associated HCC. The tetraspanin CD81 is an essential receptor for HCV, however, its role in hepatoma biology is uncertain. We demonstrate that antibody engagement of CD81 promotes hepatoma spread, which is limited by HCV infection, in an actin-dependent manner and identify an essential role for the C-terminal interaction with Ezrin-Radixin-Moesin (ERM) proteins in this process. We show enhanced hepatoma migration and invasion following expression of CD81 and a reduction in invasive potential upon CD81 silencing. In addition, we reveal poorly differentiated HCC express significantly higher levels of CD81 compared to adjacent non-tumor tissue. In summary, these data support a role for CD81 in regulating hepatoma mobility and propose CD81 as a tumour promoter.

## 1. Introduction

Tetraspanins are a family of widely expressed small membrane proteins that share common structural features: four transmembrane domains, a small intracellular loop, two short intracytoplasmic termini, and two extra-cellular loops (EC1 and EC2). These membrane proteins establish multiple interactions with themselves and other transmembrane or cytosolic proteins to form tetraspanin-enriched microdomains (TEMs) that recruit and regulate signaling molecules. Tetraspanins are considered to be “molecular organizers” by regulating associated signaling proteins via lateral protein-protein interactions. Their role(s) vary depending on the cell type and tetraspanin in question but have been reported to include cell adhesion, proliferation, motility and signal transducing processes frequently associated with cancer progression (for reviews see [1,2,3]). Some tetraspanins including CD9, CD82 and CD63 are considered to be tumour suppressors whereas others such as CD151 and CO-029 promote metastasis. Tetraspanins have been suggested as prognostic markers for cancer progression and potential therapeutic targets (for review see [4,5]).

The tetraspanin CD81 was originally identified as the target protein for an antibody that inhibited lymphocyte proliferation [6] and more recently as a receptor defining hepatitis C virus (HCV) infection of hepatocytes [7,8]. Antibody engagement of CD81 on immune cells promotes actin cytoskeletal rearrangement and altered cellular function including: T cell stimulation via activation of Lck a Src family protein kinase [9,10]; inhibition of NK cytolytic activity [11,12] and B-cell activation and proliferation [13,14,15]. Given the role of hepatocellular CD81 in HCV infection and the association of chronic hepatitis C with hepatocellular carcinoma (HCC) there is a growing interest in studying the function of CD81 in hepatocytes. Antibody ligation of CD81 on Huh-7 hepatoma cells has been reported to activate the MAPK-ERK and Rho-family GTPase signaling pathways [16,17,18]. Itakura and colleagues reported CD81 genetic polymorphisms in HCC tissue, suggesting a role for CD81 in hepatocarcinogenesis [19]. In contrast, Mazzocca and colleagues reported that CD81 suppressed hepatoma motility *in vitro* [20]. Given the limited and conflicting nature of these reports we investigated the role of CD81 in hepatoma biology.

We demonstrate that antibody engagement of CD81 induces hepatoma spread in an actin-dependent manner and identify a role for the *C*-terminus and Ezrin-Radixin-Moesin (ERM) proteins in this process. Bivalent antibody ligation of CD81 is essential to promote the changes in hepatoma morphology, consistent with antibody induced cross-linking of CD81. HCV infection perturbs CD81-dependent hepatoma spread that may have important consequences for HCV associated HCC pathogenesis. CD81 expression promoted hepatoma invasion and migration, supporting a role for CD81 in hepatoma motility. Furthermore, silencing CD81 expression in Huh-7 hepatoma cells attenuated their invasive potential in vitro. Importantly, we demonstrate an association between CD81 expression in HCC that is dependent on the degree of tumor differentiation, where poorly differentiated HCC express significantly higher levels of CD81 compared to the adjacent non-tumor tissue. In summary, our data support a role for CD81 in regulating hepatoma mobility and implicate CD81 as a tumour promoter.

## 2. Results

### 2.1. CD81 Engagement Promotes Actin-Dependent Hepatoma Spread

**Figure 1:**
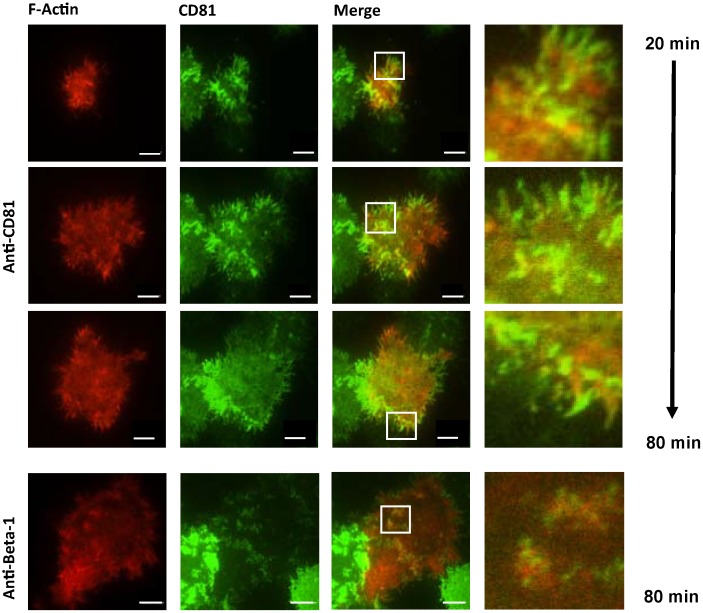
CD81 and F-actin expression in Huh-7.5 hepatoma cells. Huh-7.5 cells, engineered to express AcGFP.CD81 (green) and Ruby tagged LifeAct (red), were seeded onto glass bottom 35 mm dishes, coated with anti-CD81 (1s262) or anti-Beta-1. Images were collected by total internal reflection fluorescence (TIRF) microscopy every 10 min for a total of 80 min. Images depict CD81-Actin co-localization over time during cell adhesion and spread on anti-CD81 or anti-Beta-1 coated surfaces. Overlap coefficient R = 0.93 Anti-CD81 and R = 0.94 Anti-Beta-1 at 80 min; the data set is representative of two independent experiments. Scale bar represents 10 µm.

To assess whether antibody engagement of CD81 promotes actin polymerization and alters hepatoma morphology, Huh-7.5 cells were transfected to express AcGFP.CD81 and Ruby tagged LifeAct, a fluorophore labeled peptide that binds F-actin allowing live cell imaging of the actin cytoskeleton [21]. We previously reported that AcGFP.CD81 supports HCV entry [22] and localizes at the plasma membrane in a comparable fashion to untagged endogenous protein. AcGFP.CD81 and LifeAct-Ruby expressing Huh-7.5 cells were seeded onto glass dishes pre-coated with monoclonal antibodies (mAbs) specific for CD81 or Beta-1 Integrin whose ligation is well recognized to promote cell spread. The cells were imaged by total internal reflection fluorescence microscopy (TIRF) to image the cell membrane in contact with the glass-tethered antibody. Both anti-CD81 and anti-Beta-1 integrin antibodies promoted hepatoma spread in a time dependent manner (Figure 1), whereas cells failed to attach or spread on isotype control IgG coated plates. CD81 co-localized with actin in both anti-CD81 and anti-Beta-1 treated cells, suggesting that their co-localization is independent of anti-CD81 priming.

**Figure 2:**
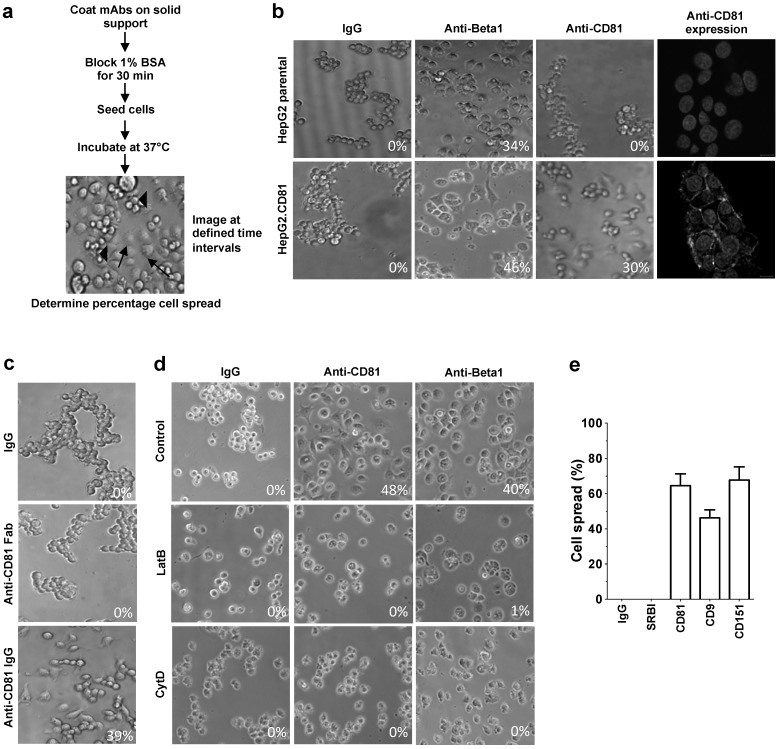
CD81 engagement promotes hepatoma spread. Cartoon depicting cell spread assay. Spreading cells (arrows) and non-spreading cells (arrow heads) are annotated (**a**). Confocal images of CD81 expression (1s262, 5 µg/mL) in HepG2 cells, phase images of parental HepG2 or HepG2.CD81 spread following IgG control (5 µg/mL), anti-CD81 (1s262, 5 µg/mL) or anti-Beta-1 (5 µg/mL) ligation for 6 h (**b**). Phase images of HepG2.CD81 cell spread following IgG control (5 µg/mL), anti-CD81 Fab or whole IgG (2s66, 5 µg/mL) ligation for 6 h (**c**). Phase images of HepG2.CD81 spread following treatment with Cytochalasin D, Latrunculin B or control DMSO in response to IgG control (5 µg/mL), anti-CD81 (1s262, 5 µg/mL) or anti-Beta1 (5 µg/mL) (**d**). Quantification of Huh-7.5 spread following anti-SRBI, anti-CD81, anti-CD9, anti-CD151 or isotype control IgG (5 µg/mL) ligation for 1 h (**e**). Data presented are from an individual experiment representative of at least three independent experiments and include quantification of cell spread expressed as a percentage.

To study the processes involved in anti-CD81 induced hepatoma spread we adapted our assay to measure antibody-induced changes in hepatoma morphology by phase microscopy (Figure 2a). HepG2 cells, which naturally lack CD81, provide an ideal tool to evaluate CD81 specific changes in hepatoma morphology. Initial experiments determined the optimal time for anti-CD81 induced changes in cell morphology: 1 h for Huh-7.5 and 6 h for HepG2 cells stably expressing CD81 (HepG2.CD81). CD81 localized at the plasma membrane of HepG2.CD81 cells and expression promoted hepatoma spread on anti-CD81 coated wells (Figure 2b), whereas cells failed to attach or spread on isotype control IgG coated plates (Figure 2b). HepG2 cells incubated on anti-Beta-1 integrin showed a spreading phenotype irrespective of CD81 expression (Figure 2b). These results confirm the specificity of CD81 ligation induced hepatoma spread. Several mAbs specific for linear and conformation-dependent CD81 EC2 epitopes promoted HepG2.CD81 spread with comparable efficiency (Table 1). In contrast, a Fab fragment of anti-CD81 clone 2s.66 failed to promote HepG2.CD81 spread, suggesting that changes in hepatoma morphology are dependent on antibody bivalency and CD81 cross-linking (Figure 2c). The actin cytoskeleton plays an essential role in cell shape and motility [23]. To ascertain whether the changes in HepG2 shape were dependent on actin polymerization the cells were treated with Latrunculin B or Cytochalasin D that bind actin and prevent polymerization or inhibit filament elongation, respectively. Both treatments ablated the spread of HepG2.CD81 cells in response to anti-CD81, demonstrating an actin polymerization-dependent process (Figure 2d). No morphological changes were observed in the presence of an isotype control IgG and, actin modulation also inhibited anti-Beta-1 induced cell spread. We confirmed that anti-CD81 promoted the spread of Huh-7.5 hepatoma cells that naturally express CD81 (Figure 2e). To control for the potential effect of antibody tethering of cells to the plate surface inducing cell morphological changes, we demonstrate that a mAb specific for scavenger receptor BI that is expressed on Huh-7.5 cells had no effect on hepatoma morphology (Figure 2e). Furthermore, antibodies specific for tetraspanins CD9 and CD151 induced Huh-7.5 spread (Figure 2e), suggesting that this property is common amongst other tetraspanins.

**Table 1:** Monoclonal antibodies used in the study. Panel of anti-CD81 mAbs: clone ID; recognition of linear peptide epitope (NLCPSGSNIISNLFKEDCHQ); binding to HepG2.CD81 cells; cell spread in HepG2.CD81 cells.

Anti-CD81 Clone	Epitope	HepG2.CD81 antibody binding, 5 µg/mL (MFI)	Cell Spread, 5 µg/mL (%)
2s20	Linear	165	34
2s66	Linear	159	40
2s131	Linear	135	48
1s135	Conformational	69	37
1s262	Conformational	48	51
1s337	Conformational	119	30

### 2.2. CD81 Ligation Promotes Hepatoma Spread in a MAPK-Independent Manner

**Figure 3:**
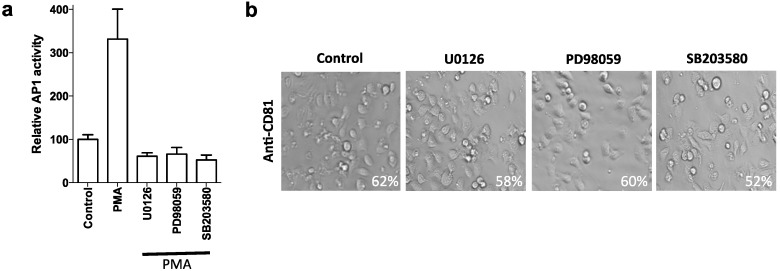
AP1 activity was measured in PMA stimulated (50 ηg/mL, 24 h) Huh-7.5 cells in the presence of MAPK inhibitors U0126, PD98059, SB203580 or DMSO control (100 µM, 24 h) (**a**). Phase images of Huh-7.5 cells following anti-CD81 (1s262, 5 µg/mL) ligation for 1 h (**b**) pre-incubated with MAPK inhibitors (100 µM, 1 h). Data presented are from an individual experiment representative of at least two independent experiments and include quantification of cell spread expressed as a percentage.

Mitogen activated protein kinase (MAPK) regulates macrophage, fibroblast and endothelial cell spread [24,25,26]. Since antibody engagement of CD81 has been reported to activate MAPK [9,10,17], we investigated the effect of MAPK inhibitors on anti-CD81 primed Huh-7.5 spread. MAPK inhibitors U0126, PD98059, and SB203580 were functionally active and reduced the levels of AP-1 transcriptional activity following phorbol ester activation of the MAPK signaling in Huh-7.5 cells (Figure 3a). None of the anti-CD81 antibodies listed in Table 1 induced AP1 activity in Huh-7.5 cells and the inhibitors had no effect on anti-CD81 induced hepatoma spread (Figure 3b). Comparable results were obtained with HepG2.CD81 cells suggesting that the process is independent of MAPK signaling.

### 2.3. CD81 C-Terminus and ERM Proteins Regulate Hepatoma Spread

**Figure 4:**
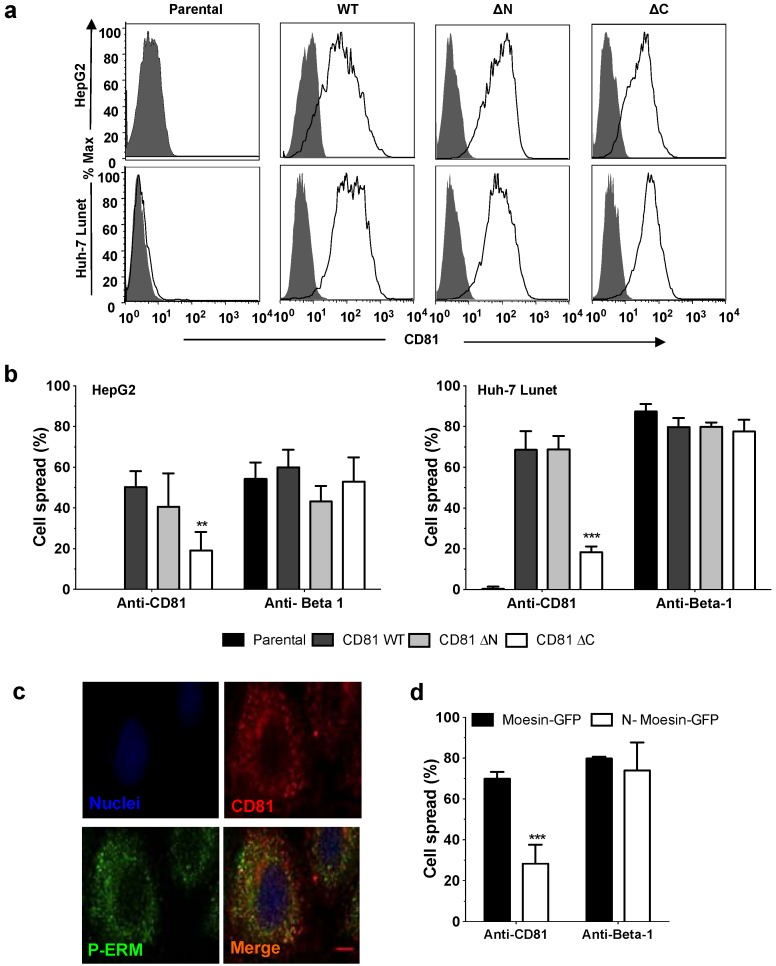
CD81 *C*-terminal-dependent hepatoma spread and role of actin-associated Ezrin-Radixin-Moesin (ERM) in CD81-dependent hepatoma spread. Flow cytometric detection of CD81 (2s131, 5 µg/mL) in HepG2 or Huh-7 Lunet cells transduced to express non-tagged CD81_WT_, CD81_ΔN_ or CD81_ΔC_, where filled histogram depicts irrelevant IgG control (**a**). Percentage spread of HepG2 or Huh-7 Lunet cells transfected to express CD81_WT_, CD81_ΔN_ or CD81_ΔC_ following anti-CD81 (1s262) or anti-Beta-1 (5 µg/mL) ligation for 6 h (HepG2.CD81) or 1 h (Huh-7 Lunet) (**b**). Confocal images depicting CD81 co-localization with phosphorylated ERM proteins in Huh-7.5 cells (**c**). Percentage spread of Huh-7.5 cells transfected to express Moesin-GFP or the dominant negative N-Moesin-GFP following anti-CD81 (1s262, 5 µg/mL) or anti-Beta-1 (5 µg/mL) ligation for 1 h (**d**). Data presented are from an individual experiment representative of at least three independent experiments, *** *p* ≤ 0.001.

The CD81 *C*-terminus has been reported to regulate protein dynamics and viral receptor activity [15,27,28]. To determine whether the *C*-terminus plays a role in hepatoma spread wild type (wt) or mutant CD81 lacking either the *N*- or *C*-termini (CD81_ΔN_ or CD81_ΔC_) were expressed in HepG2 parental cells, that are devoid of endogenous CD81 expression, or Huh-7 Lunet, a sub-clone that express negligible CD81 (Figure 4a). HepG2 and Huh-7 Lunet expressing CD81_ΔC_ show significantly reduced spread following anti-CD81 engagement compared to cells expressing wt or CD81_ΔN_. In contrast, all cells respond to anti-Beta 1 integrin engagement (Figure 4b). Actin-associated ERM proteins link the CD81 *C*-terminus to the actin cytoskeleton [27]. Phosphorylation of ERM proteins is important for actin rearrangement (for review see [29]) and confocal imaging demonstrates CD81 co-localization with phospho-ERM in unstimulated cells (Pearson’s co-localization coefficient, Rr = 0.662 ± 0.047) (Figure 4c). To determine whether ERM proteins play a role in anti-CD81 induced hepatoma spread, Huh-7.5 cells were transduced to express a GFP tagged dominant negative Moesin unable to bind F-actin (N-Moesin-GFP) or control Moesin-GFP [30,31]. Cells expressing N-Moesin-GFP showed significantly reduced anti-CD81 spread (Figure 4d), but no effect on anti-Beta1 primed changes in cell morphology. In summary, these data highlight a role for the CD81 *C*-terminus and ERM proteins in promoting CD81-dependent hepatoma spread. 

### 2.4. HCV Infection Perturbs CD81-Dependent Hepatoma Spread

**Figure 5:**
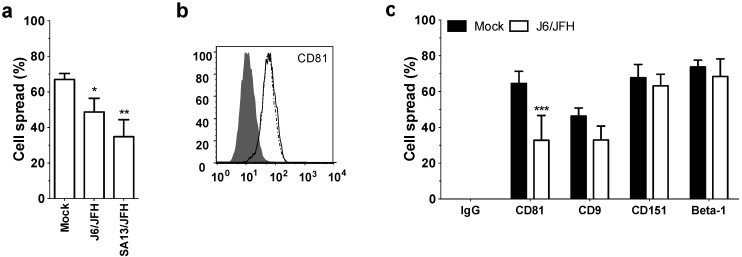
Hepatitis C virus (HCV) infection perturbs CD81-dependent hepatoma spread. Percentage spread of naïve or HCV J6/JFH (63%, NS5A positive) or SA13/JFH (73%, NS5A positive) infected Huh-7.5 cells following anti-CD81 (1s262, 5 µg/mL) ligation for 1 h (**a**). Flow cytometric detection of CD81 expression (1s262, 5 µg/mL) in naïve (dotted line) and J6/JFH infected (solid line) Huh-7.5 cells, where the filled histogram depicts an irrelevant isotype matched IgG control (**b**). Percentage spread of naïve and HCV J6/JFH (63%, NS5A positive) infected Huh-7.5 cells following 1h ligation with IgG control, anti-CD81 anti-CD9, anti-CD151 or anti-Beta-1 (5 µg/mL) (**c**). Data presented are from an individual experiment representative of at least three independent experiments, * *p* ≤ 0.05, ** *p* ≤ 0.01, *** *p* ≤ 0.001.

Given the recent report that HCV infection reduces moesin expression [32] we were interested to study the effect of HCV infection on CD81-dependent hepatoma spread. Infection of Huh-7.5 hepatoma cells with HCV strains J6/JFH and SA13/JFH led to a significant reduction in CD81-dependent hepatoma spread (Figure 5a). Since many viruses are known to down regulate their receptors to prevent super infection [33,34], we quantified CD81 expression level on naïve and HCV infected cells by flow cytometry and observed comparable protein expression (Figure 5b). To determine if this phenomenon was common for other tetraspanins and associated proteins, we compared the spread of naïve and HCV infected Huh-7.5 cells following ligation with antibodies targeting tetraspanins CD9 and CD151 or associated protein integrin Beta-1. We confirmed reduced spread of HCV infected cells with anti-CD81 (Figure 5c). In contrast, naïve and infected hepatoma cells showed comparable spread following stimulation with anti-CD9, anti-CD151 or -Beta-1 integrin (Figure 5c).

### 2.5. Role of CD81 in Hepatoma Motility and Adhesion

**Figure 6:**
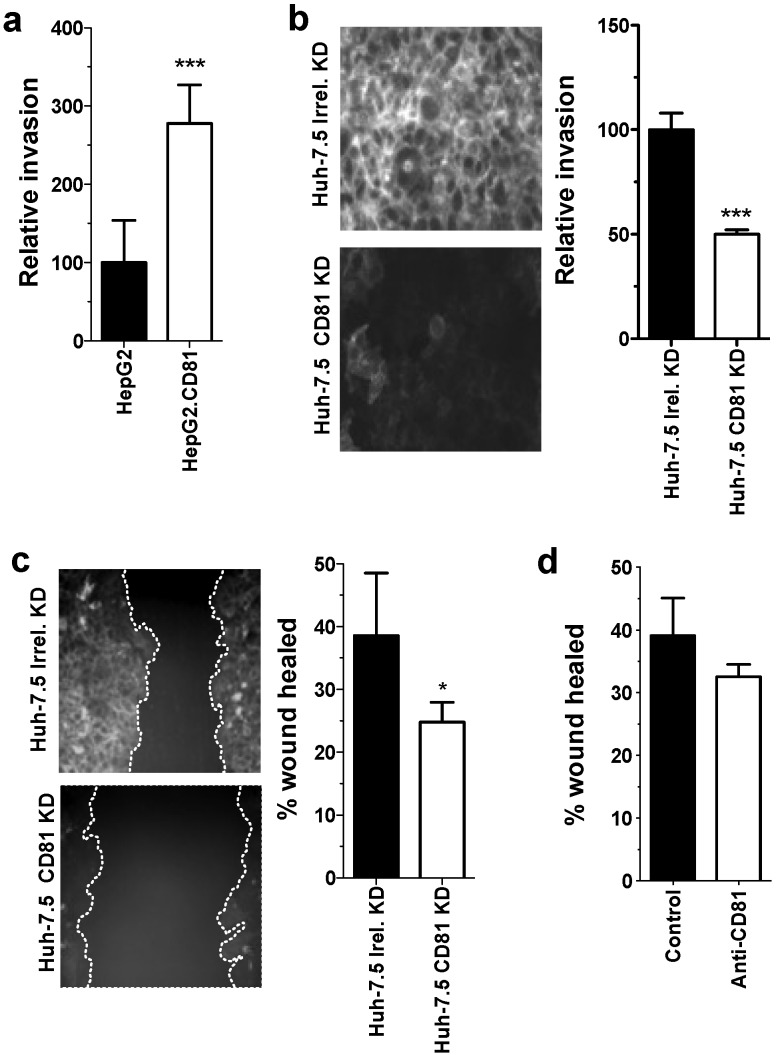
CD81 expression promotes hepatoma cell invasion and migration. Invasion of (**a**) parental or CD81 expressing HepG2; (**b**) naïve and CD81 siRNA transfected Huh-7.5 cells through collagen over 24 h. Immunofluorescence images of CD81 expression in naïve and CD81 siRNA tranfected Huh-7.5 cells (**b**). Phase images and analysis of wound healing in (**c**) naïve and CD81 siRNA tranfected Huh-7.5 cells; (**d**) IgG control or anti-CD81 (1s262, 5 µg/mL) stimulated Huh-7.5 cells. Data presented are from an individual experiment representative of at least three independent experiments, * *p* ≤ 0.05, *** *p* ≤ 0.001.

To investigate whether CD81 promotes hepatoma motility, we assessed the ability of HepG2 and Huh-7.5 cells to invade collagen I extracellular matrix and to migrate in a scratch wound assay, features that are considered to define hepatoma metastatic potential *in vivo*. Ectopic expression of CD81 promotes HepG2 invasion (Figure 6a). siRNA silencing of CD81 expression in Huh-7.5 cells reduced their invasive (Figure 6b) and migratory capacity (Figure 6c). Given our previous observation that anti-CD81 ligation promotes hepatoma spread, we measured the effect of anti-CD81 mAb on Huh-7.5 migration in the scratch wound assay. No differences were observed in wound closure in antibody treated or untreated cells (Figure 6d). These findings support a role for CD81 in hepatoma invasion and migration, suggesting a positive regulatory role for CD81 in hepatoma metastasis.

### 2.6. CD81 Expression in Hepatocellular Carcinoma Tissue

**Figure 7:**
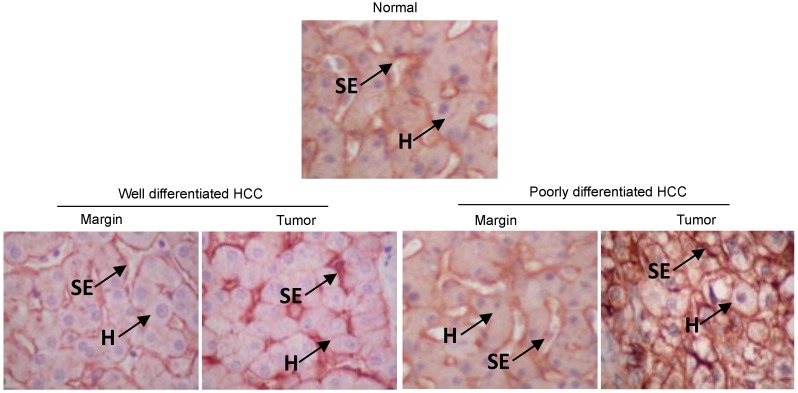
CD81 expression is associated with tumour differentiation status. Representative immunohistochemical staining of CD81 (2s131, 5 µg/mL) in normal liver, peri-tumoral tissue and hepatocellular carcinoma (HCC) tumor tissue with well and poorly differentiated HCC (n = 5 in each category). CD81 expression on the sinusoidal endothelium (SE) or hepatocyte (H) membrane is shown, ×200 magnification.

Formalin fixed paraffin embedded tissue samples from normal and diseased tissue were sectioned and stained for CD81 expression. CD81 was predominantly expressed on the sinusoidal endothelium in normal liver tissue consistent with previously published reports [35] (Figure 7). A similar expression pattern was observed in cirrhotic tissue adjoining tumor. However, neoplastic hepatocytes in HCC expressed higher levels of CD81 and this was more marked in poorly-differentiated tumour compared to well-differentiated HCC (Figure 7).

## 3. Discussion

Our study highlights a role for CD81 in promoting the invasion and motility of hepatoma cell lines. We demonstrate that antibody ligation of CD81 promoted hepatoma spread in an actin-dependent manner, identifying a role for the intracellular *C*-terminal CD81 domain and its association with the actin cytoskeleton via ERM proteins. These *in vitro* observations are supported by *ex vivo* staining of liver tissue showing increased CD81 expression in poorly differentiated tumor tissue compared to adjacent non-tumor areas, implicating CD81 as a tumor promoter. Our data contrasts to a previous report showing that CD81 expression reduced HepG2 migration [20], however, we note that the majority of CD81 localized to intracellular vesicles with minimal expression at the plasma membrane. We previously reported that CD81 is largely expressed at the hepatocellular plasma membrane with elevated levels at cell-cell contacts [36], suggesting an atypical localization in the Mazzocca study.

The majority of published anti-tetraspanin antibodies bind the second extracellular loop (EC2) that mediates tetraspanin-tetraspanin and tetraspanin-partner protein interactions [37]. We show that a number of antibodies targeting linear and conformational CD81 epitopes in EC2 induce hepatoma spread, suggesting that this phenotype is independent of epitope or antibody binding affinity. To ascertain whether ligation of other cell surface proteins induce hepatoma spread we evaluated antibodies specific for other tetraspanin family proteins (CD9 and CD151) and the non-tetraspanin cell surface expressed protein scavenger receptor B1 (SR-B1). Antibodies targeting SR-B1 had no effect whereas the spreading phenotype was observed following stimulation of CD81 associated tetraspanins CD9 and CD151. CD151 is known to be a promoter of metastasis and several antibodies targeting this tetraspanin have been reported to inhibit tumour spread, motility and invasion *in vitro* and *in vivo* [38]. With the exception of platelets CD81 is ubiquitously expressed and is not considered a therapeutic target, nevertheless a recent study demonstrated that intravenous administration of an anti-CD81 mAb in cynomolgus monkeys leads to a defined accumulation in the liver [39]. Although our studies have identified a role for CD81 in hepatoma cell migration and invasion, anti-CD81 mAbs failed to modulate these processes suggesting limited therapeutic potential.

The CD81 *C*-terminus plays a role in anti-CD81 induced effects on immune cell phenotype [15,27]. Hepatoma cells expressing CD81_ΔC_ displayed a significantly reduced ability to spread following CD81 ligation, suggesting an important role for the *C*-terminus in hepatoma morphology. The *C*-terminus of CD81 contains a PDZ binding domain that associates with ERM proteins [27], where ERM phosphorylation regulates the crosslinking of actin filaments to integral membrane proteins. Activation of ERM proteins is driven by threonine phosphorylation (Ezrin Thr567, Radixin Thr564, and Moesin Thr558) [40,41,42], and previous reports have shown increased P-ERM in anti-CD81 stimulated NK and B cells [15,43]. We failed to detect any significant increase in P-ERM following anti-CD81 ligation of hepatoma cells, however, this is likely due to elevated P-ERM expression in these cells (Figure 4c) that may mask changes following CD81 ligation. However, hepatoma cells expressing N-Moesin-GFP, a dominant negative mutant lacking the *C*-terminal actin-binding domain [30], showed a reduced ability to spread in response to CD81 ligation. A recent report showing that HCV infection reduces the expression of ERM proteins moesin and radixin [32] provides an explanation for our observation that HCV infection limits CD81-dependent hepatoma spread. We recently reported a role for the CD81 *C*-terminus of CD81 and its association with the actin cytoskeleton via ERM proteins in CD81 lateral diffusion at the plasma membrane [28], supporting a dynamic interplay of CD81 with partner proteins and the actin cytoskeleton in defining hepatoma mobility.

The reduced spread of CD81-primed hepatoma cells following HCV infection demonstrates an indirect role for HCV to modulate hepatoma spread or metastasis. We previously reported that HCV promotes hepatoma invasion via stabilizing hypoxia inducible factor-1a and increased vascular endothelial growth factor expression [44], demonstrating the multifactorial nature of pathways underlying hepatoma metastasis and highlighting a positive regulatory role for CD81 in HCC pathogenesis. Matrix metalloproteinases (MMP) play a significant role in tumour invasion and metastasis (for review see [45]) and CD81 has been reported to associate with MT1-MMP [46,47,48]. Furthermore, MT1-MMP/MMP-14 expression is increased in the HCV infected liver and has been reported to associate with disease progression [49]. Our observation that increased CD81 expression associates with tumour grade may be indicative of increased MT1-MMP expression in poorly differentiated tumours.

## 4. Experimental Section

### 4.1. Cell Culture, Cell Lines and Reagents

Huh-7.5 (Charles Rice, Rockefeller University, New York, NY, USA), Huh-7 Lunet (Thomas Pietschmann, Twincore, Hannover, Germany) and HepG2 cells were propagated in Dulbecco’s modified medium (DMEM) with 10% fetal bovine serum (FBS), 1% non-essential amino acids and maintained in a humidified atmosphere at 37 °C and 5% CO_2_. Anti-CD81 mAbs (Table 1), were generated following immunization of mice with recombinant full-length CD81 [28,36,50]; anti-NS5A (9E10) (Charles Rice, Rockefeller University, New York, NY, USA); anti-phospho-ERM (Cell Signaling Technologies, MA, USA); anti-Beta-1 integrin (P5D2, R&D systems, Minneapolis, MN, USA); anti-scavenger receptor BI (SRBI) (MAb PF72 a gift from Pfizer Ltd., New York, NY, USA); anti-CD151 (11B1) and anti-CD9 (IAA2) [51] (Michael Tomlinson, University of Birmingham, Birmingham, UK). Species-specific Alexa Fluor 488 or 594, IgG were purchased from Invitrogen (Carlsbad, CA, USA). pRTM3 plasmids encoding Moesin-GFP and the dominant negative N-Moesin-GFP [30] (Maria Yanez-Mo, Instituto de Investigación Sanitaria Princesa, Madrid, Spain) were transfected into cells using Lipofectamine 2000 (Invitrogen) according to the manufacturer’s guidelines. Cells were used 48 h post transfection. Lentiviruses expressing AcGFP.CD81 [22], wild type CD81 (CD81_WT_), CD81_ΔN_ and CD81_ΔC_ lacking their *N*- or *C*-terminal tails, respectively, were used to transduce hepatoma cells as previously reported [36] (Martin Hemler, Dana-Farber Cancer Institute, Boston, MA, USA). Cells were transfected with LifeAct-Ruby using Lipofectamine 2000 according to manufacturers instructions and used 24 h post transfection. HCV infections were initiated as previously described [52,53]. Briefly, HCV RNA J6/JFH or SA13/JFH was transcribed *in vitro* from full-length genomes (RiboMax t7 kit, Promega, Madison, WI, USA) and electroporated into Huh-7.5 cells. Supernatants containing virus were harvested at 72 and 96 h post-infection, pooled, and stored at −80 °C. Virus was used to infect naïve Huh-7.5 cells for 72 h, cells fixed and infected cells detected by staining for HCV encoded NS5A. Bound antibody was detected with anti-mouse IgG Alexa-488 and quantified by enumerating NS5A expressing cells by flow cytometry (BD Biosciences, San Jose, CA, USA).

### 4.2. Total Internal Reflection Fluorescence (TIRF) Imaging

Huh-7.5 cells were transduced to express AcGFP.CD81 [22] and subsequently transfected with Ruby-tagged LifeAct. Cells were seeded on glass bottom dishes coated with antibodies (5 µg/mL) and serum starved overnight prior to assay. Cells were imaged, using an oil immersion objective at ×100 magnification and 100 nm depth of field, over the defined time period by TIRF microscopy (Nikon A1R, Amsterdam, Netherlands). Intracellular colocalization was determined using ImageJ colocalization finder plug-in software [54], and the overlap coefficient presented.

### 4.3. Hepatoma Spread

Cells were serum starved overnight and where indicated pre-treated with Cytochalasin D (5 µM, Sigma, Dorset, UK) or Latrunculin B (0.1 µM, Sigma) at 37 °C for 1 h, mitogen activated protein kinase (MAPK) inhibitors U0126, PD98059, SB203580 (100 µM, Calbiochem, Darmstadt, Germany) at 37 °C for 24 h. Antibodies (5 µg/mL) were bound to non-tissue culture treated plates onto which cells were seeded and incubated at 37 °C for 6 h (HepG2) or 1 h (Huh-7.5, Huh-7 Lunet) in the presence of inhibitors. Cells were imaged by phase microscopy (Nikon TE2000), the total number of cells in a field of view enumerated and the percentage of cells showing a spreading phenotype indicated by a flattening morphology presented as mean ± SD. Statistical analyses were performed using the student t test in Prism 6.0 [55].

### 4.4. Immunofluorescence

Cells were cultured on 13mm-diameter borosilicate glass cover slips prior to fixing with electron microscope grade formaldehyde (EM-F) (TAAB, Aldermaston, Berkshire, UK). Following permeabilization cells were incubated sequentially with primary and secondary antibodies. Actin was visualized using Phalloidin-594 (Sigma), cells counterstained with 4',6'-diamidino-2-phenylindole (DAPI) (Invitrogen) and coverslips mounted onto glass slides (ProLong Gold antifade; Invitrogen). Cells were imaged by laser scanning confocal microscopy using a 63× water immersion objective (Zeiss LSM510, OberKochen, Germany).

### 4.5. Flow Cytometric Analysis of Cell Surface Protein Expression

Cells were incubated in FACS buffer (2%BSA/0.01%Azide/PBS) prior to incubating with primary antibody or irrelevant isotype control and detection with Alexa-fluor 488 species-specific secondary antibody. Cells were fixed (1% PFA) and cell surface protein expression determined by flow cytometry (BD Biosciences). 

### 4.6. Determination of MAPK Activity

Hepatoma cells were transfected, using Lipofectamine 2000 (Invitrogen) in accordance with the manufacturer’s instructions, with an AP-1-luciferase reporter construct (Michael Tomlinson, University of Birmingham, Birmingham, UK), in which expression of luciferase is under the control of the MAPK pathway. 4 h after transfection cells were incubated with the MAPK activator phorbol-12-myristate-13-acetate (PMA, 50 ηg/mL, Sigma, Dorset, UK) or inhibitors (100 µM) of MAPK for 24 h in appropriate medium. Cells were lysed and luciferase activity quantified in a luminometer (Berthold Centro LB 960, Berthold, Bundoora, Australia). The amount of luciferase detected is an indicator of MAPK activity and defined as arbitary Relative Light Unit (RLU) relative to untreated control cells.

### 4.7. Hepatoma Invasion

Hepatoma cells were serum starved overnight prior to assay set up. For assays using CD81 knock down cells, cells were transfected with CD81 or irrelevant control siRNA (Invitrogen) using Lipofectamine RNAiMAX (Invitrogen) and used 24 h post transfection. 4 × 10^4^ viable cells were seeded into the top chamber of a collagen (calf collagen, Sigma) coated transwell (Falcon 8 µM pore, 24 well plate); the bottom chamber of the transwell contained serum free DMEM. After 24h incubation at 37 °C, non-migratory cells were mechanically removed with a cotton bud. Migrated cells were fixed with ice-cold methanol, incubated for 1hr with 0.1% crystal violet and then washed with PBS. Three fields of view per well were captured on a Nikon TE2000 fluorescence microscope. Invaded cells were enumerated and the data presented as relative invasion compared to control ± SD. Statistical analyses were performed using the student t test in Graphpad Prism 6.0 [55].

### 4.8. Hepatoma Scratch Wound

Huh-7.5 cells were serum starved overnight prior to assay set up. For assays using CD81 knock down cells, cells were transfected with CD81 or irrelevant control siRNA (Invitrogen) using Lipofectamine RNAiMAX (Invitrogen) and used 24 h post transfection. A wound was performed using a P200 tip, and cells incubated at 37 °C for the defined time under serum free conditions in the presence or absence of soluble 1s262 (5 µg/mL). Images were taken on a Nikon TE2000 fluorescence microscope at 0 and 24 h post wounding. The wound area was quantified using IPLab 4.0 software (BD Biosciences) [56]; the data is presented as mean percentage wound healed ± SD. Statistical analyses were performed using the student t test in Prism 6.0 [55].

### 4.9. Liver Tissue and Immunohistochemistry

Formalin-fixed paraffin embedded specimens of tumor and surrounding non-neoplastic liver tissue were obtained from 10 patients who underwent liver transplantation at the Queen Elizabeth Hospital Birmingham, UK for HCC complicating cirrhosis of non-viral aetiology. 5 patients had well differentiated HCC (grade 1) and 5 had poorly differentiated tumors (grade 3). Liver disease was diagnosed according standard clinical, histological and radiological criteria. Normal liver tissue was obtained from surplus donor tissue (n = 5) used for reduced size transplantation with patient consent and regional ethical committee approval obtained. Representative 5 μM sections were cut from paraffin blocks and placed onto slides; sections were de-waxed with Clearene (Leica Biosystems, Milton Keynes, Buckinghamshire, UK) and rehydrated followed by treatment with an endogenous peroxidase (Dako, Glostrup, Denmark). Tissue was subjected to low temperature epitope retrieval and sections incubated with anti-CD81 primary antibody (2s131 [In house]) diluted in tris-buffered saline/0.1% Tween-20 (TBS/Tween) for 1 h. After a TBS/Tween wash sections were visualized with ImmPACT DAB Chromogen (Vector Laboratories, Cambridgeshire, UK). Two observers including an experienced liver pathologist (SGH) performed microscopic examination. 

## 5. Conclusions

In summary, our data illustrate a role for CD81 in regulating hepatoma migration and invasion, which may impact on HCC metastasis. These conclusions are supported by our *ex vivo* studies demonstrating an association between histological tumour grade and CD81 expression. Poorly differentiated HCCs are known to have an increased capacity for invasion, including microvascular invasion, leading to the development of metastases [57,58,59]. Studies of patients undergoing liver resection or transplantation for HCC have shown that high tumour grade is an important risk factor for tumour recurrence and, consequently, for poor survival [57,60]. These observations increase our understanding of hepatocellular carcinoma and may have important implications for the study of viral associated and non-associated hepatocellular carcinoma, an increasing global health burden [61]. 
